# Zinc Status of Horses and Ponies: Relevance of Health, Horse Type, Sex, Age, and Test Material

**DOI:** 10.3390/vetsci10040295

**Published:** 2023-04-16

**Authors:** Sarah van Bömmel-Wegmann, Heidrun Gehlen, Ann-Kristin Barton, Kathrin Büttner, Jürgen Zentek, Nadine Paßlack

**Affiliations:** 1 Institute of Animal Nutrition, Freie Universität Berlin, 14195 Berlin, Germany; sarah.wegmann@fu-berlin.de (S.v.B.-W.); juergen.zentek@fu-berlin.de (J.Z.); 2 Equine Clinic, Freie Universität Berlin, 14163 Berlin, Germany; heidrun.gehlen@fu-berlin.de (H.G.); ann-kristin.barton@fu-berlin.de (A.-K.B.); 3 Unit for Biomathematics and Data Processing, Justus-Liebig-University Giessen, 35392 Giessen, Germany; kathrin.buettner@vetmed.uni-giessen.de

**Keywords:** zinc, diet, equines, plasma, mane hair, age, sex

## Abstract

**Simple Summary:**

Little is known about the effects of animal- and diet-related factors on the plasma zinc (Zn) concentrations of horses and ponies. Additionally, it is unclear if plasma samples are adequate to reflect changes in the Zn intake. In the first part of the study, the impact of age, sex, horse type (ponies vs. horses), and internal diseases on the plasma Zn concentrations was evaluated in 538 patients of an Equine Clinic. The second part was a feeding study with two healthy horses and eight healthy ponies to assess the effects of two dietary Zn supplements on the plasma and mane hair Zn concentrations of the animals. Part 1: The age, sex, and horse type did not influence the plasma Zn concentrations. No effect of internal diseases was observed, with the exception of higher plasma Zn concentrations in animals with metabolic diseases compared to the control group. Part 2: Both Zn supplements increased the Zn concentrations in the mane hair, but not in the plasma, in a dose-dependent manner. In conclusion, the plasma Zn concentrations were widely unaffected by nutritional and non-nutritional factors in horses and ponies, while mane hair samples better reflected the Zn supply with the diet.

**Abstract:**

Little is known about the animal- and diet-related factors that could interfere with the plasma zinc (Zn) concentrations of equines. Additionally, the adequacy of plasma to reflect changes in the Zn intake is unclear. In the first part of this study, the plasma Zn concentrations of hospitalized horses and ponies (*n* = 538) were measured and evaluated for the impact of the age, sex, horse type, and internal diseases of the animals. In the second part, the effects of increasing dietary Zn chloride hydroxide and Zn methionine supplementations were assessed on the plasma and mane hair Zn concentrations of healthy horses (*n* = 2) and ponies (*n* = 8). Part 1: The age, sex, and horse type did not influence the plasma Zn concentrations. No effect of internal diseases was observed, with the exception of higher plasma Zn concentrations in animals with metabolic disorders compared to the control group (*p* < 0.05). Part 2: Both Zn supplements dose-dependently increased the Zn concentrations in the mane hair (*p* = 0.003), but not in the plasma of the horses and ponies. In conclusion, the plasma Zn concentrations were widely unaffected by nutritional and non-nutritional factors in equines, while mane hair samples better reflected the dietary Zn supply.

## 1. Introduction

Zinc (Zn) is an essential component of several enzymes in the organism and involved in a wide range of physiological functions, thus playing an important role for biological processes and health [1,2]. While horses seem to tolerate relatively high amounts of dietary Zn, a Zn deficiency has been demonstrated to result in skin and hair disorders, reduced growth, and inappetence [3].

In order to evaluate the Zn status of horses and ponies, blood samples are often collected. However, blood measurements imply considerable limitations, especially due to the homeostatic regulation of the body to maintain physiological plasma Zn concentrations [4]. Nevertheless, even physiological Zn levels might vary depending on individual factors, which could be practically relevant for the interpretation of the measured blood values. Human studies have indicated, although not consistently, that the age or sex of individuals can affect the serum Zn concentrations [5,6,7,8]. A few investigations in horses have also evaluated this dependency, but they could not find sex- or age-related effects in most cases [9,10,11,12,13]. Another factor that might potentially affect the plasma Zn concentrations in equines are specific diseases, which could interfere with the Zn metabolism. So far, decreased serum Zn concentrations have only been detected in horses with shipping fever, fever, and cellulitis [13], but detailed studies in hospitalized equines are missing.

Besides the consideration of those animal-related factors, it is questionable whether the dietary Zn supply can be reflected by plasma analyses. In a study by Cymbaluk and Christensen [14], higher plasma Zn concentrations of yearling horses were not always associated with the highest Zn intakes. To our best knowledge, further targeted studies on the dietary Zn supply and the related plasma Zn concentrations of horses are missing so far.

In place of serum or plasma analyses, the measurement of the Zn concentrations in hair samples could be interesting to assess the Zn status of horses and its potential influencing factors. Hair is advantageous compared to other biological test material, since it is easy to collect and without significant demands for storage [15]. It might also reflect the mineral status of an animal more reliable, but only for the longer term, as it is metabolically relatively inert [16], and incorporates minerals only during the follicle formation [17], i.e., before keratinization and reaching the skin surface [15]. On the other hand, several non-nutritional factors are discussed to influence the mineral concentrations in hair, making direct conclusions on the relationship to the mineral intake of an individual partly difficult. For the Zn concentrations in the hair of horses, effects of the age [18], sex [19,20], or breed [18] of the animals, as well as of the hair color [21] have been described, although contradicting data also exist [15,22,23].

Only two studies have evaluated the impact of a dietary Zn supplementation on the mane hair Zn concentrations of horses so far. The results of Armelin et al. [24] demonstrated that the intake of a mineral supplement affected the Zn, iron, and potassium concentrations in the mane hair of horses; however, the authors constrained that several minerals were supplemented in combination, thus they were unable to comment on their single nutritional effects [24].

Ghorbani et al. [19] also used a dietary supplement, which was composed of different minerals. The mane hair Zn concentrations were significantly higher in the horses with the highest Zn intake compared to the other groups, while the serum Zn concentrations were unaffected by the dietary treatments [19].

Other studies did not prove the reliability of mane hair analyses to reflect the dietary Zn intake of horses. Van der Merwe et al. [25] could not detect a correlation between the mane hair and liver Zn content of equines. The authors argued that the liver is the preferred tissue for the evaluation of the trace element status [26], while the missing correlation between the measured minerals in the mane hair and liver samples indicates an inadequacy of hair analyses for this purpose [25]. The study of Wahl and Vervuert [27] demonstrated variations in the trace element concentrations of the same hair sample in dependence on the analyzing laboratory. Moreover, since different reference ranges were considered by the laboratories, the interpretation of the nutritional status of the horses by the mane hair analyses also varied [27].

Overall, the Zn status of horses and ponies is important for equine health. Although blood samples are often used for Zn measurements, hair analyses might be also interesting. However, targeted, dose-dependent studies concerning the effects of Zn supplements on the hair Zn concentrations of equines are currently missing. With regard to the plasma Zn concentrations of horses and ponies, animal-related, interfering factors have been barely explored so far. It was therefore the aim of the present investigation to evaluate the impact of non-nutritional and nutritional factors on the Zn concentrations in equine plasma and mane hair samples in more detail.

## 2. Materials and Methods

### 2.1. Approval

In the first part of the study, the Zn concentrations were measured in plasma samples of horses and ponies hospitalized at the Equine Clinic of the Freie Universität Berlin. As the blood was collected for diagnostics, and only residual plasma was used for the present investigation, no approval of the local regulatory authority was required. However, the use of residual plasma for research purposes was communicated with the authority, and received the file number StN 0012-18 (Landesamt für Gesundheit und Soziales, Berlin, Germany).

The second part of the study was a feeding trial, which was approved by the relevant authority in Munich, Germany (ROB-55.2-2532.Vet_02-19-14).

### 2.2. Animals, Housing, and Feeding Regimen

Part 1: A total of 538 plasma samples of hospitalized horses (*n* = 384) and ponies (*n* = 154) were collected from November 2018 to March 2020. The animals were divided into a group with internal diseases (*n* = 317) and a control group with orthopedic problems (*n* = 221). The internal disease patients were further divided into the following subgroups: animals with diseases of the gastrointestinal tract (*n* = 188), respiratory tract (*n* = 40), eyes (*n* = 22), skin (*n* = 4), and metabolism (*n* = 21), as well as animals with further diseases (*n* = 42). A questionnaire on the feeding and housing regimen of the horses and ponies was additionally distributed to the owners; however, due to a low compliance, no data analysis was possible for these variables.

Part 2: Eight private-owned ponies and two private-owned horses, permanently housed in the same stable, were included in this study [28]. The animals received the following basic diet, where the daily amount was calculated to maintain their body weight (BW): grass hay (8 kg/horse, 2.5–4.5 kg/pony), crimped oats (1 kg/horse, 0.1–0.5 kg/pony), muesli (1 kg/horse, 0.1–0.5 kg/pony), and a vitamin and mineral supplement (35 g/horse, 13–35 g/pony). No Zn was added to the muesli and vitamin and mineral supplement by the manufacturers. The ration was based on the previous feeding regimen of the animals and offered throughout the experiment without modifications in the daily amounts. The dry matter intake (DMI) was constant, since the ration was completely ingested by the horses and ponies. The calculated Zn concentration of the basic diet (=without a Zn supplement) was 22.9 mg/kg DM. The horses and ponies underwent six feeding periods of four weeks each. In the first feeding period, the basic diet was fed with a supplement based on Zn chloride hydroxide to cover the maintenance Zn requirement of the animals (4 mg/kg BW^0.75^/day [29], which corresponded to a mean dietary Zn concentration of 67 mg/kg DM). In the second and third feeding period, the basic diet was supplemented with Zn chloride hydroxide to achieve 120 mg Zn/kg DMI and 240 mg Zn/kg DMI, respectively. For the fourth feeding period, a Zn supplement based on Zn methionine instead of Zn chloride hydroxide was added to the basic diet to cover the Zn maintenance requirement of the horses and ponies (4 mg/kg BW^0.75^/day [29]; corresponding to a mean Zn concentration of 67 mg/kg DM), and in the fifth and sixth feeding period, the Zn methionine supplement was combined with the basic diet to achieve 120 mg Zn/kg DMI and 240 mg Zn/kg DMI, respectively. The horses and ponies received the same Zn supplement at the same time, i.e., no cross-over design was used for this study.

### 2.3. Sample Collection

Part 1: The blood samples of the hospitalized horses and ponies were collected by veterinarians of the Equine Clinic of the Freie Universität Berlin, using tubes coated with potassium EDTA. After centrifugation of the blood at 2000× *g* for 10 min and at room temperature (Rotofix 32 A, Hettich Lab Technology, Tuttlingen, Germany), the plasma was collected for diagnostic purposes. If plasma was left after the diagnostic measurements, it was stored at −20 °C and could be used for the Zn measurements of the present study.

The EDTA tubes were routinely used in the Equine Clinic to obtain plasma for diagnostic purposes. Since only residual plasma was analyzed for the present investigation, no additional blood tube could be provided for the blood collection. As it has been described that EDTA can chelate erythrocytic Zn, which may result in a contamination of the plasma [30], the use of tubes with another anticoagulant might have been preferable. However, recent studies could demonstrate no difference between the plasma Zn concentrations, when EDTA or lithium heparin tubes were used for the blood collection [31,32], and also a significant [32] or close to significant [31] correlation between the EDTA and lithium heparin plasma Zn concentrations. Moreover, as the EDTA tubes were used for the entire study population of the present investigation, a potential interfering factor of the anticoagulant would be the same for all samples and should therefore not bias the results.

Part 2: In the morning of the last day of each feeding period, blood and mane hair samples of the horses and ponies were collected. The blood was collected in lithium heparin tubes. For the mane hair sampling, a strand of hair was cut as closely as possible to the withers of the animals by using ceramic scissors. Due to the slow hair growth during the 4-week feeding periods, it was not possible to use only the newly grown hair for the Zn analyses. Instead, a new strand of hair was cut at the end of each feeding period. The strands were of 1 cm width and equally trimmed from their low ends to a length of 10 cm. The hair was stored in sterile polypropylene tubes until further analysis.

### 2.4. Zn Measurements

For the measurement of the Zn concentrations in the plasma samples, 200 µL plasma were mixed with 100 µL hydrochloric acid (w = 10%) in a first step. After incubation at room temperature for 10 min, 160 µL of a trichloroacetic acid solution (w = 10%) and 1 mL cesium chloride–lanthanum chloride buffer (10 g cesium chloride/L + 100 g lanthanum chloride/L) were added. The samples were centrifuged for 10 min at room temperature and 2500× *g*, and the supernatant was pipetted into a polypropylene tube for the consecutive Zn measurements. For this, a flame atomic absorption spectrometer (FAAS) (Analytik Jena AG, Jena, Germany) and the software ASpect CS 2.2 (Analytik Jena AG, Jena, Germany) were used.

The mane hair samples were washed multiple times with a Zn-free dish detergent and ultrapure water to remove adherent dirt particles. Afterwards, the hairs were dried for at least 4 h in a ventilated compartment dryer at 60 °C, and ashed in a muffle furnace at 600 °C over night. The samples were mixed with 7 mL ultrapure water and 2.1 mL concentrated hydrochloric acid (37–38%), and heated in a sand bath at 220 °C for 1 h. After cooling, the solution was transferred through a pleated filter into a 10 mL graduated flask, which was then filled up with distilled water. Afterwards, the solution was treated as described for the plasma samples, and the Zn concentrations were measured by FAAS.

### 2.5. Establishment of a Reference Range

As reference intervals can vary, among others depending on the method used for the sample analysis [33], a reference range for the plasma Zn concentrations of the hospitalized horses and ponies was established based on the obtained data set. For this, the program Reference Value Advisor (Version 2.1) was used. Since the sample size was ≥120, a non-parametric method of the program could be used for the reference interval calculation, which is in accordance with current guidelines [34].

### 2.6. Statistical Data Analysis

The data of the first part of the study were analyzed using SAS (SAS^®^ 9.4, 2nd edition, Statistical Analysis System Institute Inc., Cary, NC, USA) and SPSS 28 (IBM Corp., Armonk, NY, USA). As the Shapiro–Wilk test demonstrated that the data were not normally distributed, non-parametric tests were used for the following analyses. The groups of the hospitalized horses and ponies were compared by the Kruskal–Wallis and Mann–Whitney U-test, and the Spearman’s rank correlation coefficient was used for the evaluation of potential dependencies between the plasma Zn concentrations and the age of the animals. The data are presented as median (minimum–maximum). A *p*-value < 0.05 was considered to be statistically significant.

For the second part of the study, the data were analyzed with SPSS 26 (IBM Corp., Armonk, NY, USA). The data were normally distributed, as indicated by the Shapiro–Wilk test. A repeated-measures ANOVA with Greenhouse–Geisser corrected *p*-values was used to detect the effects of the within-subject factors “dietary Zn dose” (three levels) and “dietary Zn compound” (two levels). If a significant interaction occurred between the two within-subject factors, polynomial contrasts were calculated for each Zn compound. In the case that the interaction was not significant, the data for both Zn compounds were analyzed together. Linear and quadratic polynomial contrasts were then calculated for the Zn dose, and linear polynomial contrasts for the Zn compound. Although the data were normally distributed, they are presented in the table as median (minimum–maximum) to acknowledge the individual variability of the Zn concentrations in biological samples, as demonstrated in the first part of the study with a larger study population. Group differences were considered to be statistically significant at a *p*-value < 0.05. A trend for a group effect was determined at 0.05 < *p* < 0.10.

## 3. Results

### 3.1. Plasma Zn Concentrations of the Hospitalized Horses and Ponies (Study Part 1)

#### 3.1.1. Calculated Reference Range

The calculated reference range for the plasma Zn concentrations of the hospitalized horses and ponies is presented in Table 1. Comparable reference values were found for the total study population, the control group, and the horses and ponies with internal diseases.

#### 3.1.2. Dependence on the Health Status of the Animals

The median plasma Zn concentrations did not differ between the control animals and the horses and ponies with internal diseases (*p* = 0.344), and were within the established reference range (Table 1). When the internal disease patients were subdivided depending on their diagnoses, the median plasma Zn concentrations of these groups were also within the reference range (Table 2). The plasma Zn concentrations were higher in the horses and ponies with metabolic diseases compared to the control animals (*p* < 0.05).

#### 3.1.3. Correlation with the Age of the Animals

**Figure 1:**
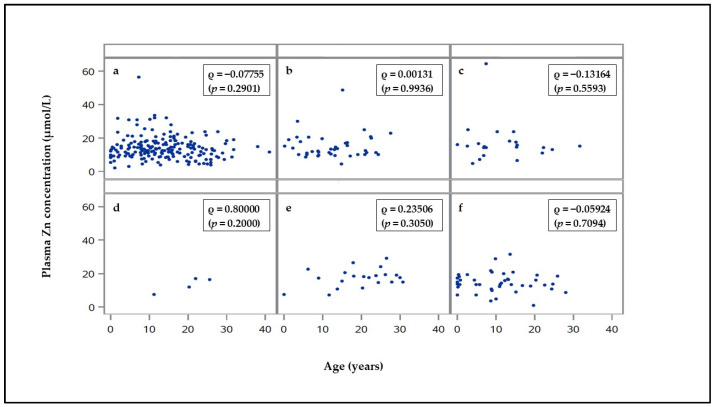
Correlation between the plasma Zn concentration and the age of the horses and ponies with (**a**) gastrointestinal diseases (*n* = 188), (**b**) respiratory diseases (*n* = 40), (**c**) eye diseases (*n* = 22), (**d**) skin diseases (*n* = 4), (**e**) metabolic diseases (*n* = 21), and (**f**) further diseases (*n* = 42). Spearman’s rank correlation coefficient ρ (*p*-value).

The age of the hospitalized horses and ponies, divided into the control animals and the subgroups of the internal disease patients, is provided as a Supplementary Table S1. The groups differed in the age of the animals, but the correlation analysis revealed no dependency of the plasma Zn concentration on the age (specified in years) of the total study population (Spearman´s rank correlation coefficient: ρ = 0.02367; *p* = 0.5838), of the control group (ρ = 0.08311; *p* = 0.2185), the internal disease patients (ρ = −0.00742; *p* = 0.8953), or of the subgroups with internal diseases (Figure 1).

#### 3.1.4. Dependence on the Sex of the Animals

No dependency could be detected between the plasma Zn concentrations and the sex of the horses and ponies, neither when analyzing the total study population, nor the control and internal disease patients separately (*p* > 0.05) (Table 3; Supplementary Table S2).

#### 3.1.5. Dependence on the Horse Type

For the evaluation of the dependence of the plasma Zn concentrations on the horse type, the total study population was divided either in “ponies” and “horses” (category 1) or in “ponies”, “cold-blooded horses”, “warm-blooded horses”, and “thoroughbred horses” (category 2) (Supplementary Table S3). However, due to the irregular distribution of the animals in category 2, only category 1 was statistically analyzed. No dependency was observed between the horse type and the plasma Zn concentrations of the total population, the control group, or the internal disease patients (*p* > 0.05) (Table 4; Supplementary Table S4).

### 3.2. Zn Concentrations in the Mane Hair and Plasma of Healthy Adult Horses and Ponies (Study Part 2)

Increasing dietary Zn concentrations increased the Zn content in the mane hair of the horses and ponies, independently of the dietary Zn compound (linear contrast for dose, *p* = 0.003) (Table 5). There was only a trend for slightly higher median mane hair Zn concentrations, when Zn chloride hydroxide instead of Zn methionine was included in the diet (*p* = 0.074).

With regard to the plasma concentrations, only a quadratic effect was detected for the supplementation of Zn methionine, with the lowest plasma Zn concentration being when the horses and ponies received the medium Zn methionine dose (*p* < 0.001).

## 4. Discussion

In the present study, nutritional and non-nutritional factors that could affect the Zn status of horses and ponies were evaluated. We did not detect an impact of the age or sex of the animals on the plasma Zn concentrations, which is in line with most studies in equines [10,11,12,13]. Only the study of Cymbaluk et al. [9] could demonstrate age dependencies in young horses. The Zn concentrations in the plasma of newborn and one-week-old Standardbred–Thoroughbred horses were higher than in yearlings and adult Standardbred–Thoroughbred horses [9]. However, no differences in the plasma Zn concentrations were observed between draft-cross neonates and their dams [9].

In human medicine, contradictory data exists. While some studies could demonstrate higher serum Zn concentrations in men [5,8] or women [7], or age-dependent effects [6,8], other authors could not find sex- or age-related differences [6,35,36,37,38,39,40]. Age and sex effects on the serum Zn concentrations are potentially explained by differences in the serum albumin concentrations, which also vary depending on the sex and age of human subjects [41]. As most of the Zn in the blood circulation is bound to albumin, changes in the serum albumin levels might also affect the serum Zn concentrations [41]. Another potential explanation for the observed sex-related variations in the serum Zn levels could be differences in the lean body mass, which is correlated with the exchangeable Zn pool masses in the human organism [42]. In the present study, no information was available on the serum albumin concentrations or the body condition score of the hospitalized horses and ponies; therefore, no relationships with the plasma Zn concentrations could be explored. Nevertheless, our data could confirm previous findings that the age and sex of horses and ponies were not interfering with their plasma Zn concentrations.

Little is known about the impact of the health status of horses and ponies on the plasma Zn concentrations. Two studies with Icelandic horses could not detect a difference between animals with dermatitis (*Culicoides* hypersensitivity, sweet itch) and a healthy control group [10,12]. In the investigation of Stark et al. [10], a negative correlation between the plasma Zn concentrations and the severity of the *Culicoides* hypersensitivity was observed. However, due to the small changes, the authors questioned the clinical relevance of this finding [10]. In an experimentally induced acute-phase reaction by the intramuscular injection of Freund’s adjuvant, the plasma Zn concentrations decreased in horses [43], indicating that inflammation might impact the Zn status of equines. In line, Murase et al. [13] could demonstrate that the serum Zn concentrations were lower in horses with shipping fever, fever, and cellulitis compared to control animals. However, except for the horses with shipping fever, all values were within the estimated reference range [13]. The authors also found that the serum Zn concentrations of horses experimentally infected with *Streptococcus equi* subsp. *zooepidemicus* decreased below the reference value the day after infection [13].

In the present study, the horses and ponies with internal diseases were divided into six subgroups. Only the animals with metabolic disorders showed higher plasma Zn concentrations than the control group, while no differences were observed among the horses and ponies with intestinal, respiratory, ocular, skin, or further diseases. Different diagnoses were summarized in the subgroup with metabolic disorders (Supplementary Table S5); however, 52% of the patients were affected by pituitary pars intermedia dysfunction (PPID). To our best knowledge, no data on altered plasma or serum Zn concentrations in horses with PPID have been published so far. In a field study by Kienzle and Bockhorni [44], the dietary Zn concentrations for horses with PPID were often below the recommendations of the GfE [29]. One might hypothesize that a dietary oversupply with Zn could therefore be unlikely for the observed higher plasma Zn concentrations in the horses and ponies with metabolic disorders, particularly with PPID. However, dietary patterns vary widely for equines, and it is a limitation of the present study that no information on the feeding regimen was available for the hospitalized patients. Thus, the data interpretation is difficult at this stage. It should also be mentioned that the plasma Zn values of the animals with metabolic diseases were within the established reference range, and that the sample size of this subgroup was relatively small (*n* = 21). Thus, the clinical relevance of the higher plasma Zn levels in the horses and ponies with metabolic diseases is uncertain, but warrants further investigation in future studies.

The comparison between the hospitalized horses and ponies of the present study did not reveal differences in the plasma Zn concentrations depending on the horse type. Due to the irregular distribution within the study group, we could not evaluate variations in the plasma Zn concentrations between ponies, cold-blooded, warm-blooded, and Thoroughbred horses, nor could we investigate potential breed differences. In the study of Cymbaluk et al. [9], draft-cross horses had the highest plasma Zn concentrations of the breeds investigated. Other available studies on the plasma or serum Zn concentrations of equines generally included horses of different breeds [10,11,12,13,14,19], but targeted comparisons on their blood Zn levels are widely missing and require further investigation.

For the interpretation of the present data, it should finally be considered that the samples of the hospitalized horses and ponies were collected for a period of more than one year, and that the patients of the Equine Clinic mainly included horses and ponies from Berlin and the surrounding area. As previous investigations have indicated seasonal or geographical influences on the plasma or serum Zn concentrations of equines, the present study design might imply certain limitations. In the investigation of Gromadzka-Ostrowsk et al. [45], the highest plasma Zn concentrations of Shetland pony mares were observed in January. The authors discussed a potential role of the pineal gland for these seasonal fluctuations, especially with regard to horses in reproduction, as both the pineal gland and the reproductive function are activated, when the days are becoming longer [45]. Kolm et al. [12] could detect differences in the plasma Zn concentrations of Icelandic horses depending on their housing farms. The same observation was published by Stark et al. [10]. Potential influencing factors on the farms included the diet, housing, environment, and management [10], which were, however, not further evaluated in this study. Finally, Ali et al. [46] demonstrated that the serum Zn concentrations varied depending on the reproductive phase and housing conditions of mares. In the present investigation, it can be assumed that several factors, such as the feeding and housing management, season, and location, have influenced the plasma Zn concentrations of the hospitalized horses and ponies. As these factors have not been explored as a part of this study, correlation analyses were not possible. However, regardless of potential interfering external factors, the large sample size (*n* = 538) allowed for a general analysis of the effects of the animal-related factors, such as age, sex, horse type, and health status, on the plasma Zn concentrations of equines. Overall, the impact of these factors was small, indicating a metabolic regulation of the plasma Zn concentrations in horses and ponies.

Based on this assumption, further biological test material might be required to assess the Zn status of equines, with mane hair being an interesting option. In the second part of the present study, we compared the effects of increasing dietary Zn doses on the plasma and mane hair Zn concentrations of horses and ponies. It could be proved that the dietary Zn supply did not clearly affect the plasma Zn concentrations. Although high Zn doses were evaluated, the plasma Zn levels remained unchanged, with the exception of a quadratic effect for the Zn methionine supplementation. It should be noted, however, that no dietary Zn deficiency was evaluated in this study, and that all plasma Zn values were within the established reference range, even at very high dietary Zn intakes.

In contrast to the plasma Zn concentrations, the mane hair analyses revealed a linear effect of the increasing dietary Zn doses. The horses and ponies of this study were of different breeds, sexes, ages, and hair colors. These factors have been discussed to affect the Zn concentrations in equine mane hair [18,19,20,21], although other authors could not support this assumption [15,22,23]. However, as the present study group was consistent, i.e., as all horses received both supplements, potential animal-related factors influencing the mane hair Zn concentrations can be neglected. Nevertheless, the mane hair measurements of this study imply limitations, since the hair growth was not sufficient to analyze only the newly grown hair. According to Whittem et al. [47], a mean hair growth rate of 0.059 cm per day can be assumed, which corresponds to approximately 1.5 cm for our 4-week feeding periods. As the mane hair samples were ashed prior to the Zn measurements, the amount of the newly grown hair was not adequate to run these analyses. Thus, complete strands of hair were cut at the end of each feeding period. Since not only the newly grown hair, but also parts of the older hair were therefore analyzed, the measured Zn concentrations were not only ascribed to the latest feeding period, but also to the preceding ones. However, as the strands were equally trimmed to a length of 10 cm, the proportion of the “new” and “old” hair was similar among all horses and ponies. Moreover, since the animals received the same dietary treatment in all feeding periods, i.e., since no cross-over-design was used, the impact of the preceding feeding periods was also the same throughout the study. Thus, the differences between the median Zn concentrations in the mane hair of the horses and ponies can be considered reliable to reflect the differences in the dietary Zn intake, although the measured total Zn concentration does not equate to the amount of Zn that was stored in the mane hair during only one single feeding period. Overall, it can be concluded that the dietary Zn supply was better reflected by the Zn concentrations in the mane hair than in the plasma of the horses and ponies. It should, however, be noted that the differences in the mane hair Zn concentrations were relatively small among the feeding groups, although the dietary Zn intakes markedly differed. In addition, the small sample size of the second study part requires a careful data interpretation.

Interestingly, the effects on the mane hair Zn concentrations were comparable between the two Zn supplements evaluated in the present study. Organic and inorganic Zn sources are often assumed to have a different bioavailability [48,49], which might affect the hair Zn concentrations as well. However, both the inorganic Zn chloride hydroxide and the organic Zn methionine dose-dependently increased the Zn concentrations in the mane hair of the horses and ponies of the present investigation. Our previous data could partly demonstrate divergent compound effects of these Zn supplements on the fecal microbiota [28] and the immune system [50] of the animals. These results might indicate a different intestinal absorption and metabolic utilization of Zn chloride hydroxide and Zn methionine in equines [28]. The mane hair analyses, however, revealed only a trend for the Zn compound. Thus, both compounds seemed to be well absorbed in the intestine of the horses and ponies, as demonstrated by the small, but dose-dependent effects on the mane hair Zn concentrations.

## 5. Conclusions

The plasma Zn concentrations of horses and ponies were only marginally affected by animal- and health-related factors. In contrast to plasma, mane hair samples better reflected a dietary Zn supplementation, although the group differences were relatively small. Overall, our data imply that this test material might be potentially more adequate to assess the Zn status of equines. Future studies with a larger study population would be interesting to confirm this assumption.

## Figures and Tables

**Table 1:** Lower and upper reference limits for the plasma Zn concentrations (µmol/L) of the hospitalized horses and ponies, as well as the measured values (µmol/L; median (minimum–maximum)) of the total study population and the two main subgroups.

Study Population	*n*	Lower Reference Limit	Upper Reference Limit	Measured Plasma Zn Concentration ^1^	
Total population	(538)	4.40	31.2	14.1	(0.82–64.3)
Control group	(221)	5.60	30.4	14.3	(2.98–63.0)
Patients with internal diseases	(317)	4.30	31.3	13.8	(0.82–64.3)

^1^ No significant difference was found between the control group and the horses and ponies with internal diseases, *p* = 0.344.

**Table 2:** Plasma Zn concentrations (µmol/L) of the control group and the subgroups of the horses and ponies with internal diseases. Median (minimum–maximum).

Study Population	*n*	Plasma Zn ^1^	
Control group	(221)	14.3	(2.98–63.0) ^a^
Patients with			
gastrointestinal diseases	(188)	13.2	(2.02–56.4) ^a^
respiratory diseases	(40)	12.6	(4.42–48.6) ^a^
eye diseases	(22)	14.9	(4.63–64.3) ^a^
skin diseases	(4)	14.1	(7.32–16.9) ^a^
metabolic diseases	(21)	17.5	(7.13–29.0) ^b^
further diseases	(42)	13.6	(0.82–31.3) ^a^

^1^ Comparison between the control group vs. the single subgroups of the internal disease patients (Mann–Whitney U-test). Not share a letter in the column indicates a significant difference between the control group vs. a subgroup of the patients with internal diseases (*p* < 0.05).

**Table 3:** Plasma Zn concentrations (µmol/L), depending on the sex of the hospitalized horses and ponies. Median (minimum–maximum).

Study Population	Sex	*n*	Plasma Zn		*p*-Value ^1^
Total population	Mare	(248)	13.9	(2.02–34.5)	0.872
	Stallion	(71)	14.3	(4.42–31.8)	
	Gelding	(219)	14.2	(0.82–64.3)	
Control group	Mare	(90)	14.5	(3.09–34.5)	0.741
	Stallion	(33)	13.9	(5.86–22.3)	
	Gelding	(98)	14.3	(2.98–63.0)	
Patients with internal diseases	Mare	(158)	13.5	(2.02–33.4)	0.769
	Stallion	(38)	14.7	(4.42–31.8)	
	Gelding	(121)	13.9	(0.82–64.3)	

^1^ Kruskal–Wallis test.

**Table 4:** Plasma Zn concentrations (µmol/L) of the hospitalized horses and ponies, depending on the horse type. Median (minimum–maximum).

Study Population	Horse Type	*n*	Plasma Zn		*p*-Value ^1^
Total population	Ponies	(154)	14.0	(3.83–56.4)	0.915
	Horses	(384)	14.1	(0.82–64.3)	
Control group	Ponies	(57)	14.3	(3.83–32.8)	0.495
	Horses	(164)	14.3	(2.98–63.0)	
Patients with internal diseases	Ponies	(97)	13.8	(4.22–56.4)	0.467
	Horses	(220)	13.7	(0.82–64.3)	

^1^ Mann–Whitney U-test.

**Table 5:** Impact of increasing doses of Zn chloride hydroxide and Zn methionine in a diet ^1^ on the Zn concentrations in the plasma and mane hair of healthy adult horses (*n* = 2) and ponies (*n* = 8). Median (minimum–maximum).

							*p*-Value (Polynomial Contrasts)							
	Zn Chloride Hydroxide			Zn Methionine			Interaction	Zn Chloride Hydroxide		Zn Methionine		Dose		Compound
	Maintenance	120 mg	240 mg	Maintenance	120 mg	240 mg		Lin	Quad	Lin	Quad	Lin	Quad	
Plasma Zn (µmol/L)	10.3	9.04	9.13	9.18	6.90	10.2	0.016	0.127	0.166	0.069	<0.001	-	-	-
	(7.57–12.6)	(8.23–10.5)	(7.80–11.5)	(6.82–11.2)	(5.05–10.0)	(7.47–14.0)								
Mane hair Zn (mg/kg)	128	126	139	125	133	135	0.057	-	-	-	-	0.003	0.292	0.074
	(92.7–145)	(83.5–139)	(102–160)	(87.8–161)	(101–158)	(115–164)								

^1^ Maintenance Zn requirement: 4 mg Zn/kg BW^0.75^/day [29]; 120 mg Zn/kg DMI; 240 mg Zn/kg DMI. Lin: linear; Quad: quadratic.

## Data Availability

The raw data of this study can be obtained from the corresponding author upon reasonable request.

## Supplementary Materials

The following supporting information can be downloaded at: https://www.mdpi.com/article/10.3390/vetsci10040295/s1, Table S1: Age of the control group and the subgroups of the patients with internal diseases. Median (minimum–maximum); Table S2: Plasma zinc (Zn) concentrations (µmol/L) of the hospitalized horses and ponies with internal diseases, depending on the sex of the animals of the six subgroups. Median (minimum–maximum); Table S3: Sample size (*n*) and percentage distribution (%) of the horse types of the present study; Table S4: Plasma Zn concentrations (µmol/L) of the hospitalized horses and ponies with internal diseases, depending on the horse type of the animals of the six subgroups. Median (minimum–maximum); Table S5: Diagnoses and plasma Zn concentrations of the horses and ponies with metabolic disorders (*n* = 21).
