# Supplementary material for: Zinc Status of Horses and Ponies: Relevance of Health, Horse Type, Sex, Age, and Test Material
Source: Vet Sci. 2023 Apr 16;10(4):295. doi: 10.3390/vetsci10040295 (PMC10144230; doi:10.3390/vetsci10040295)
Supplement: Supplementary file 1 [file vetsci-10-00295-s001.zip › vetsci-2308742-supplementary.pdf]

Supplementary Table S1: Age of the control group and the subgroups of the patients with internal diseases. Median (minimum-maximum).

Study population	*n*	Age (years)		*p*-value[1]
Control group	(221)	10.9	(0.03-38.3) [a]	< 0.001
Patients with				
gastrointestinal diseases	(188)	12.9	(0.01-41.0) [c]	
respiratory diseases	(40)	12.6	(0.20-27.6) [ac]	
eye diseases	(22)	8.85	(0.02-31.6) [ac]	
skin diseases	(4)	21.2	(11.2-25.6) [bc]	
metabolic diseases	(21)	20.6	(0.00-30.7) [b]	
further diseases	(42)	10.5	(0.00-28.0) [a]	

[1] Kruskal-Wallis test for the overall group comparison; single group comparisons by the Mann-Whitney U-test: varying exponents in the column indicate a significant group difference ($p < 0.05$)

Supplementary Table S2: Plasma zinc (Zn) concentrations (µmol/L) of the hospitalized horses and ponies with internal diseases, depending on the sex of the animals of the six subgroups. Median (minimum-maximum).

Patients with	Sex	*n*	Plasma Zn		*p*-value[1]
gastrointestinal diseases	Mare	(98)	13.3	(2.02-33.4)	0.902
	Stallion	(17)	13.9	(5.82-31.8)	
	Gelding	(73)	13.1	(3.77-56.4)	
respiratory diseases	Mare	(25)	11.5	(8.60-29.9)	0.492
	Stallion	(5)	11.8	(4.42-18.9)	
	Gelding	(10)	15.1	(9.46-48.6)	
eye diseases	Mare	(7)	14.2	(4.63-17.6)	0.240
	Stallion	(5)	15.9	(14.7-24.9)	
	Gelding	(10)	14.7	(6.42-64.3)	
skin diseases	Mare	(1)	7.32	-	0.180
	Stallion	(0)	-	-	
	Gelding	(3)	16.2	(11.9-16.9)	
metabolic diseases	Mare	(7)	18.5	(7.13-22.4)	0.904
	Stallion	(3)	14.9	(7.31-26.4)	
	Gelding	(11)	17.5	(10.8-29.0)	
further diseases	Mare	(20)	13.9	(3.72-28.9)	0.723
	Stallion	(8)	13.3	(7.27-19.2)	
	Gelding	(14)	13.4	(0.82-31.3)	

[1] Kruskal-Wallis test

Supplementary Table S3: Sample size (*n*) and percentage distribution (%) of the horse types of the present study.

	Category 1		Category 2			
**Horse type**	**Ponies**	**Horses**	**Ponies**	**Cold-blooded horses**	**Warm-blooded horses**	**Thoroughbred horses**
n	154	384	154	16	342	26
**%**	28.6	71.4	28.6	2.97	63.6	4.83

Supplementary Table S4: Plasma Zn concentrations (µmol/L) of the hospitalized horses and ponies with internal diseases, depending on the horse type of the animals of the six subgroups. Median (minimum-maximum).

Patients with	Horse type	*n*	Plasma Zn		*p*-value[1]
gastrointestinal diseases	Ponies	(54)	13.6	(4.22-56.4)	0.087
	Horses	(134)	12.6	(2.02-33.4)	
respiratory diseases	Ponies	(17)	11.5	(4.42-48.6)	0.075
	Horses	(23)	15.5	(8.60-29.9)	
eye diseases	Ponies	(3)	15.1	(14.2-15.6)	0.962
	Horses	(19)	14.7	(4.63-64.3)	
skin diseases	Ponies	(3)	11.9	(7.32-16.9)	0.655
	Horses	(1)	16.2	-	
metabolic diseases	Ponies	(11)	17.5	(11.2-26.4)	0.526
	Horses	(10)	17.6	(7.13-29.0)	
further diseases	Ponies	(9)	13.2	(7.12-19.9)	0.382
	Horses	(33)	13.7	(0.82-31.3)	

[1] Mann-Whitney U-test

Supplementary Table S5: Diagnoses and plasma Zn concentrations of the horses and ponies with metabolic disorders (*n* = 21)

Horse/pony (number)	Diagnosis	Plasma Zn (µmol/L)
1	Chronic kidney disease; Polysaccharide Storage Myopathy; hepatopathy	7.13
2	Inflammation of the urethra	10.8
3	Voiding dysfunction; inguinal hernias on both sides	7.31
4	Laminitis; acute allergic reaction (multiple edemata, disability)	11.2
5	Abscess at the preputium	14.5
6	Laminitis	22.4
7	Voiding dysfunction	19.3
8	Laminitis	17.1
9	Voiding dysfunction (sabulous cystitis)	29.0
10	Hypocalcemia (neurological symptoms)	18.0
11	Pars Pituitary Intermedia Dysfunction	24.0
12	Pars Pituitary Intermedia Dysfunction	17.5
13	Pars Pituitary Intermedia Dysfunction	18.9
14	Pars Pituitary Intermedia Dysfunction	26.4
15	Pars Pituitary Intermedia Dysfunction	20.3
16	Pars Pituitary Intermedia Dysfunction	18.5
17	Pars Pituitary Intermedia Dysfunction	18.7
18	Pars Pituitary Intermedia Dysfunction	17.4
19	Pars Pituitary Intermedia Dysfunction	15.3
20	Pars Pituitary Intermedia Dysfunction	14.7
21	Pars Pituitary Intermedia Dysfunction	14.9
